# The ERK1/2 Signaling Pathway Is Involved in Sulfur Dioxide Preconditioning-Induced Protection against Cardiac Dysfunction in Isolated Perfused Rat Heart Subjected to Myocardial Ischemia/Reperfusion

**DOI:** 10.3390/ijms141122190

**Published:** 2013-11-08

**Authors:** Pan Huang, Yan Sun, Jinyan Yang, Siyao Chen, Angie Dong Liu, Lukas Holmberg, Xiaomei Huang, Chaoshu Tang, Junbao Du, Hongfang Jin

**Affiliations:** 1 Department of Pediatrics, Peking University First Hospital, Xi-An Men Str. No. 1, West District, Beijing 100034, China; E-Mails: huangpan036@163.com (P.H.); yansun2008@gmail.com (Y.S.); yangjinyan_tg@163.com (J.Y.); siyaochen_nymaz@163.com (S.C.); hxmay111@163.com (X.H.); junbaodu1@126.com (J.D.); 2 Department of Medical and Health Sciences, Linköping University, Linköping 58183, Sweden; E-Mails: angie.dongliu@hotmail.com (A.D.L.); lukas.holmberg20@gmail.com (L.H.); 3 Key Laboratory of Molecular Cardiology, Ministry of Education, Beijing 100191, China; E-Mail: tangchaoshu@263.net.cn; 4 Department of Physiology and Pathophysiology, Health Sciences Center, Peking University, Beijing 100191, China

**Keywords:** myocardial ischemia/reperfusion injury, sulfur dioxide, preconditioning, mitogen activated protein kinase

## Abstract

Ischemia/reperfusion injury (IRI) occurs frequently during reperfusion of ischemic myocardium, and preconditioning has been regarded as one of the best strategies to prevent myocardial injury during the ischemia/reperfusion process. Our previous studies indicated that a small dose of sulfur dioxide (SO_2_) used as preconditioning exerts cardioprotection. However, the mechanisms underlying the cardioprotection remain unclear. The present study was designed to examine if the extracellular regulated protein kinases 1/2 (ERK1/2) signaling pathway mediated protection against cardiac dysfunction after SO_2_ preconditioning in isolated rat hearts subjected to ischemia/reperfusion (I/R). Langendorff heart perfusion was performed *in vitro*, where 56 male Wistar rats were randomly divided into seven groups: control group, 5 μmol/L SO_2_ group (S5), 2-(2-Amino-3-methoxyphenyl)-4*H*-1-benzopyran-4-one (PD98059) + 5 μmol/L SO_2_ (PD98059 + S5) group, PD98059 group, I/R group, 5 μmol/L SO_2_ + I/R (S5 + I/R) group and PD98059 + 5 μmol/L SO_2_ + I/R (PD98059 + S5 + I/R) group. Cardiac function and myocardial phosphorylated ERK1/2 protein were measured. We found that I/R in isolated rat heart resulted in cardiac dysfunction with a significant increase in phosphorylated ERK1/2 protein. SO_2_ preconditioning markedly suppressed phosphorylated ERK1/2 protein and improved cardiac function in isolated rat heart with I/R (*p <* 0.05). However, pre-treatment with PD98059 could prevent the above effects of SO_2_ preconditioning. In conclusion, SO_2_ preconditioning protected against cardiac dysfunction in isolated rat heart subjected to I/R via suppression of the over-activation of the ERK1/2 signaling pathway.

## 1. Introduction

Myocardial ischemia/reperfusion injury (IRI) is common in clinical practice. Thus, exploring a way for myocardial protection and investigating its mechanisms are important issues in clinical science. Preconditioning has been regarded as the best strategy to prevent myocardial injury during ischemic reperfusion processes. Previous studies on preconditioning have mainly been focusing on preconditioning using ischemia, hypoxia, metabolic suppression, endotoxin, adenosine and so forth [1–3]. Interestingly, SO_2_ has, during recent years, been discovered to have important biological effects on the cardiovascular system [4–11]. Zhang, *et al*. [12] demonstrated that exogenous SO_2_ could damage the myocardium. The outcome indicated that a small dose of SO_2_ as preconditioning exerted a significantly protective effect on myocardial injury [13]. However, the mechanisms underlying this protective effect remain unclear.

Mitogen-activated protein kinases (MAPKs) are crucial in cell signaling transduction. They contain three members: p38 MAPK, c-Jun *N*-terminal kinase (JNK) and extracellular signaling-regulated kinase (ERK). MAPKs play an important role in regulating cell function and survival [14,15]. Different family members activate various pathways by attaching to corresponding cell adhesion molecules and responding to disparate stimulation to regulate cell survival, function, growth, proliferation and differentiation. Furthermore, MAPKs are also important in regulating inflammation [16–21]. Evidence shows that the ERK1/2 signaling pathway is essential to tumorigenesis and tumor progression [22,23], and that the suppression of the excessive activation of ERK1/2 could improve vasoactivity [24]. A previous study showed a sustained activation of ERK1/2 during ischemia/reperfusion [25]. Therefore, this present study was designed to focus on this component. The phosphorylation and activation of MAP kinase (ERK1/2, p44/42MAPK) could be selectively suppressed by 2-(2-Amino-3-methoxyphenyl)-4*H*-1-benzopyran-4-one (PD98059), an organic compound inhibitor frequently used to block the activity of the ERK1/2 protein kinase [25–29]. The present study aimed at investigating whether the MAPK signaling pathway was involved in the SO_2_ preconditioning-induced protection against cardiac dysfunction in isolated rat heart subjected to ischemia/reperfusion (I/R).

## 2. Results

### 2.1. Ischemia/Reperfusion Resulted in Impaired Cardiac Function in Isolated Perfused Rat Heart

**Figure 1:**
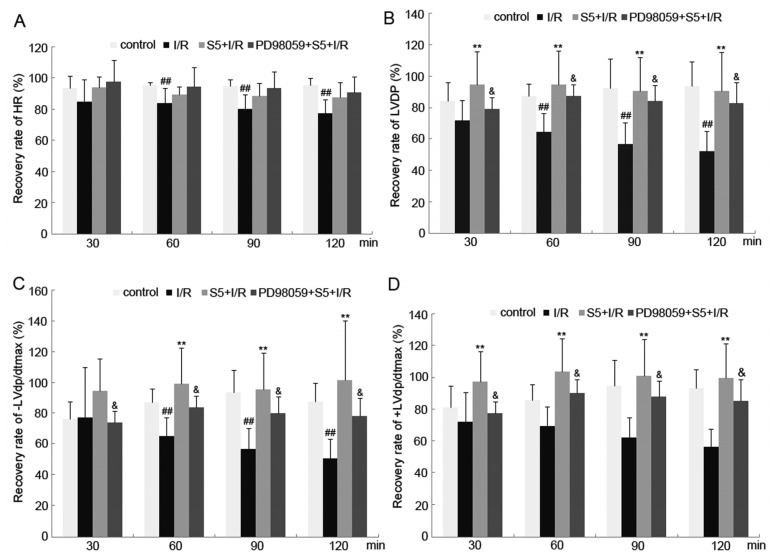
Recovery rate of heart rate (HR) (**A**); left ventricular developed pressure (LVDP) (**B**); left ventricular developed pressure maximum decreasing rate (−LVd*p*/d*t*_max_) (**C**) and left ventricular developed pressure maximum increasing rate (+LVd*p*/d*t*_max_) (**D**) of SO_2_ and 2-(2-amino-3-methoxyphenyl)-4*H*-1-benzopyran-4-one (PD98059) preconditioning on ischemia/reperfusion *in vitro* at different time points (30, 60, 90 and 120 min). Ischemia/reperfusion resulted in an impaired cardiac function in isolated perfused rat heart. SO_2_ preconditioning improved the myocardial function in isolated perfused rat heart subjected to ischemia/reperfusion, which could be abolished by treatment with PD98059. ^##^*p <* 0.01 *versus* control group; ^**^p < 0.01 *versus* ischemia/reperfusion (I/R) group; & *p <* 0.05 *versus* 5 μmol/L SO_2_ group (S5) + I/R group.

Compared with the control group, the recovery rate of heart rate (HR), left ventricular developed pressure (LVDP) and maximum decreasing rate (−LVd*p*/d*t*_max_) at 60, 90 and 120 min of ischemia/reperfusion were decreased significantly in the I/R group (*p <* 0.01) (Figure 1A–C).

### 2.2. SO_2_ Induced Phosphorylation of ERK1/2 Protein and Depressed Cardiac Function in Isolated Perfused Rat Heart without Ischemia/Reperfusion

**Figure 2:**
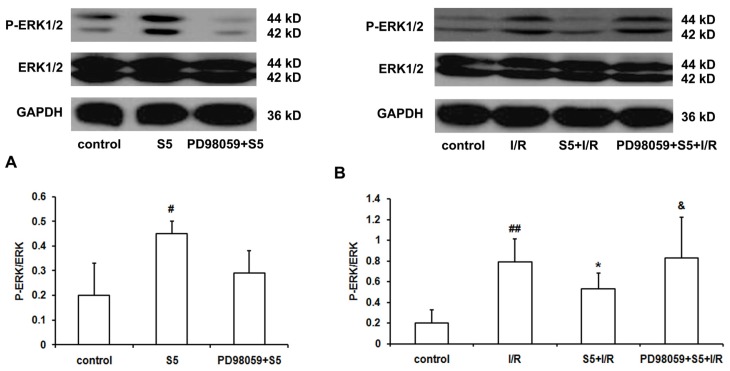
Phosphorylated ERK1/2 protein expressions of myocardium *in vitro*. SO_2_ induced phosphorylation of ERK1/2 protein in rat myocardium (**A**); SO_2_ preconditioning inhibited the excessively-induced myocardial phosphorylation of ERK1/2 protein induced by ischemia/reperfusion. Pretreatment with PD98059 successfully abolished the above inhibitory effect (**B**). ^#^*p <* 0.05; ^##^*p <* 0.01 *versus* control; ^*^p < 0.05 *versus* I/R; & *p <* 0.05 *versus* S5 + I/R.

The SO_2_ donor elevated the phosphorylation of ERK1/2 protein in rat myocardium (*p <* 0.05), which was successfully prevented by PD98059 (Figure 2A).

**Figure 3:**
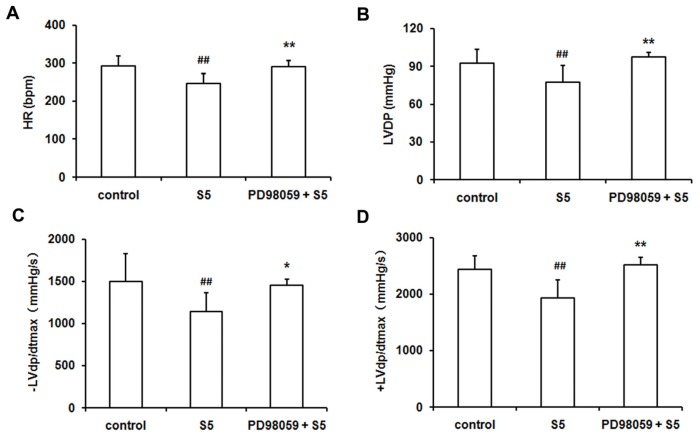
The change in HR (**A**); LVDP (**B**); −LVd*p*/d*t*_max_ (**C**) and +LVd*p*/d*t*_max_ (**D**) in the isolated rat heart without ischemia/reperfusion. The 5 μmol/L SO_2_ donor depressed the cardiac function, while PD98059 could prevent the inhibitory effect of the SO_2_ donor. ^##^*p <* 0.01 *versus* control group ^*^p < 0.05; ^**^p < 0.01 *versus* S5 group.

The SO_2_ donor depressed the cardiac function in isolated rat heart presented by the decreases in the HR, LVDP, −LVd*p*/d*t*_max_ and +LVd*p*/d*t*_max_, which was prevented by PD98059 pretreatment (Figure 3). However, PD98059 alone did not change the cardiac function in isolated rat heart (data was not shown).

### 2.3. SO_2_ Preconditioning Inhibited the Excessively-Induced Myocardial Phosphorylation of ERK1/2 Protein Induced by Ischemia/Reperfusion

In the ischemia/reperfusion group, the phosphorylation of myocardial ERK1/2 protein was significantly higher than that of the control group (*p <* 0.01). However, SO_2_ preconditioning markedly inhibited the excessively-induced myocardial phosphorylation of the ERK1/2 protein caused by ischemia/reperfusion (*p <* 0.05). However, pretreatment with PD98059 successfully abolished the above inhibitory effect (Figure 2B, *p <* 0.05).

### 2.4. SO_2_ Preconditioning Improved the Myocardial Function in Isolated Perfused Rat Heart Subjected to Ischemia/Reperfusion, Which Could Be Abolished by Treatment with PD98059

Compared with the I/R group, the recovery rate of LVDP (*p <* 0.01) and +LVd*p*/d*t*_max_ (*p <* 0.01) were increased significantly at 30, 60, 90 and 120 min of ischemia/reperfusion, respectively, for the S5 + I/R group. Furthermore, at 60, 90 and 120 min, the recovery rate of −LVd*p*/d*t*_max_ was also increased significantly (*p <* 0.01). However, the above effects could be successfully abolished by pretreatment with PD98059 (*p <* 0.05) (Figure 1B–D). The measurement of creatine kinase (CK) and glutamic-oxaloacetic transaminase (GOT) activities in the coronary perfusion fluid (CPF) showed that the increase in the CK and GOT activities of the CPF of the I/R group was alleviated by SO_2_ preconditioning (Table 1).

## 3. Discussion

I/R is an important pathophysiologic process in clinical practice and was first described by Jennings in 1960. It not only exists in different species and organs, but is involved in various pathological processes, such as multi-organ failure, shock and heart failure [30–32]. Currently, cardiovascular disease is one of the most serious threats to people’s life and health. There are now various treatments for cardiovascular diseases, such as thrombolytic therapy and coronary artery bypass grafting for coronary heart disease. Although they have decreased the mortality dramatically, the I/R injuries that come with these treatments make the results less satisfying. Therefore, the prevention and treatment of I/R are now the hot spots in medical research. The phenomenon of ischemic preconditioning was first brought out by Murry in 1986 [33], and it opened up a new field in I/R research. Thereafter, studies on IPC were conducted worldwide. However, studies about IPC have certain ethical and clinical limitations.

It has been shown that SO_2_ which is considered one of the endothelium-derived hyperpolarizing factors [34,35] can be produced endogenously from coronary arteries. Our research group previously proved that a small dose of exogenous SO_2_ could induce myocardial injury [12], and as a result, we further demonstrated that SO_2_ preconditioning could protect myocardium by antagonizing I/R *in vivo* [13]. However, the mechanisms responsible for the protection of cardiac function provided by SO_2_ preconditioning have not yet been fully understood.

MAPKs are one of the important signaling molecules in cell signal transduction. Previous studies have showed that the MEK1-ERK2 signaling pathway is involved in the regulation of cell survival [36–39]. Therefore, for the purposes of exploring the mechanisms responsible for SO_2_ preconditioning against I/R, we investigated whether the ERK/MAPK signaling pathway mediated the cardioprotection by SO_2_ preconditioning in isolated perfused rat heart subjected to I/R *in vitro*.

The results of the present study showed that the phosphorylation of ERK1/2 protein in myocardium was increased, and at the same time, cardiac function was impaired after I/R. However, SO_2_ pretreatment could elevate the phosphorylation of ERK1/2 protein in myocardiumin isolated perfused rat heart without exposure to I/R, and its preconditioning markedly inhibited the increased myocardial phosphorylation of ERK1/2 protein induced by I/R and protected impaired cardiac function and cardiac injury.

To further explore the significance of the ERK/MAPK signaling pathway in cardioprotection by SO_2_ preconditioning during I/R, we used PD98059, a MAPKK inhibitor, and observed if the cardioprotective effect of SO_2_ preconditioning could be prevented in isolated perfused rat hearts subjected to I/R. The molecular weight of PD98059 is 267.28, and its molecular formula is C_16_H_13_NO_3_. It can penetrate cells and inhibit MEK1 (one of the MAPK kinases) selectively to suppress the phosphorylation and activation of MAP kinase (ERK1/2, p44/42MAPK) [25–29]. Of note, our results showed that pretreatment with PD98059 successfully abolished the inhibitory effect of SO_2_ preconditioning on increased phosphorylation of ERK1/2 protein in the myocardium and the protective effect of SO_2_ preconditioning on the cardiac function of isolated perfused rat heart subjected to I/R. The above evidence proved that SO_2_ preconditioning could inhibit the over-activation of the ERK1/2 signaling pathway to protect the myocardium in isolated perfused rat heart during I/R.

Regarding why there was an increased ERK1/2 protein phosphorylation in the PD98059 + S5 + I/R group, we supposed that pretreatment with SO_2_ could stimulate a moderate ERK1/2 protein phosphorylation, as shown in Figure 2A. Then, in the S5 + I/R group, I/R-induced over-activation of ERK1/2 protein would be inhibited, while PD98059 inhibited ERK1/2 protein phosphorylation induced by SO_2_ pretreatment before I/R challenge, as shown in Figure 2A. Therefore, in the PD98059 + S5 + I/R group, the isolated perfused rat heart would not experience the sufficiently activated ERK1/2 status before I/R challenge. Therefore, I/R-induced over-activation of ERK1/2 protein could not be inhibited.

Our study indicated that SO_2_ preconditioning could protect myocardial function. This is possibly due to the suppression of induced ERK phosphorylation during I/R in isolated perfused rat hearts. However, the exact mechanisms by which SO_2_ preconditioning protects myocardial function from I/R need further investigation.

## 4. Experimental Section

### 4.1. Animals

Fifty-six male adult Wistar rats, weighing 250–300 g, were provided by the Experimental Animal Center, Peking University Health Science Center (Beijing, China), and had free access to water and standard rat chow. All studies were performed with the approval of the Experimental Animal Committee at Peking University, and the animals were cared for in a manner that complied with the Animal Management Rules of the Ministry of Health of the People’s Republic of China (documentation number 19890503).

### 4.2. Reagents

PD98059 was purchased from Promega (Madison, WI, USA), and p-p44/42 MAPK and p44/42 MAPK polyclonal antibodies were purchased from Cell Signaling Technology (Beverly, MA, USA). SO_2_ derivatives and a mixture of sulfite and bisulfite (Na_2_SO_3_/NaHSO_3_) in a molar ratio of 3:1 were purchased from Sigma (St. Louis, MO, USA).

### 4.3. Animal Grouping

Fifty-six male Wistar rats were divided into the following 7 groups: the control group without I/R (*n =* 8), the 5 μmol/L SO_2_ pretreated group without I/R (S5 group, *n =* 8), the PD98059 + 5 μmol/L SO_2_ pretreated group without I/R (PD98059 + S5 group, *n =* 8), the PD98059 without I/R group (PD98059 group, *n =* 8), the ischemia/reperfusion group (I/R group, *n =* 8), the 5 μmol/L SO_2_ pretreated group + I/R (S5 + I/R group, *n =* 8) and the PD98059 + 5 μmol/L SO_2_ pretreated + I/R group (PD98059 + S5 + I/R group, *n =* 8). The control group received perfusion of Krebs-Henseleit (KH) solution during the whole experiment. In the S5 group, after 20 min of stabilization, the perfusion was pretreated with KH containing 5 μmol/L SO_2_ derivatives for 5 min, and then, the rat hearts were perfused with KH solution for 5 min. In the PD98059 + S5 group, perfusion was given with PD98059 10 μmol/L for 30 min on the basis of the S5 group. In the PD98059 group, perfusion was given with PD98059 10 μmol/L for 30 min. In the I/R group, after 30 min of stabilization, perfusion was stopped for 30 min (ischemia), and the heart was then reperfused for 120 min with KH solution (37 °C). In the S5 + I/R group, 10 min before ischemia, perfusion was preconditioned with KH containing 5 μmol/L SO_2_ derivatives for 5 min. Then, the perfusion was stopped for 30 min, and then, the heart was reperfused for 120 min with KH solution (37 °C). Finally, in the PD98059 + S5 + I/R group, perfusion was given with PD98059 for 30 min before ischemia and was preconditioned with 5 μmol/L SO_2_ derivatives for 5 min, 10 min before ischemia. The heart was then reperfused for 120 min with KH solution (37 °C).

### 4.4. Heart Perfusion *In Vitro* and Cardiac Function Measurement

**Figure 4:**
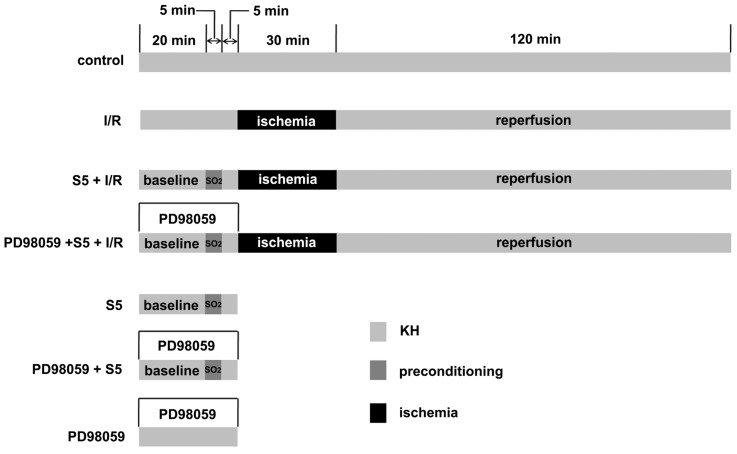
Heart perfusion procedure. Baseline: cardiac function change before ischemia. Ten min before ischemia, the rat heart was preconditioned with 5 μmol/L SO_2_ derivatives for 5 min. The heart was perfused with PD98059 for 30 min before ischemia. KH: Krebs-Henseleit.

After anesthetization with pentobarbital sodium via intraperitoneal injection at 40 mg/kg, rat hearts were taken out and placed on a HV-4 Langendorff Perfusion Apparatus (Taimeng Science and Technology Ltd., Chengdu, China) after the left auricle was cut off. Then, the rat heart was perfused with Krebs-Henseleit (KH) solution (mmol/L) [40] through the aorta retrogradely. Krebs-Henseleit (KH) solution (mmol/L) consisted of: NaCl, 118.0; KCl, 4.7; KH_2_PO_4_, 0.93; MgSO_4_·7H_2_O, 1.2; CaCl_2_, 1.5; NaHCO_3_, 25; and C_6_H_12_O_6_, 11.0 at pH 7.4 and 37 °C and with 100 cm H_2_O (1 cm H_2_O = 0.098 kPa) constant pressure and 95% O_2_/5% CO_2_ pre-saturated mixed gas. A cardiac catheter with balloon was inserted into the left ventricle from the atrioventricular valve. The balloon was filled and the left ventricular end-diastolic pressure (LVEDP) maintained 0–10 mmHg (1 mmHg = 0.133 kPa). After pre-perfusion equilibrium for 10 min, HR, LVDP and ± LVd*p*/d*t*_max_ were recorded using a BL-420F Biological Function Experiment System (Taimeng Science and Technology Ltd., Chengdu, China). The perfusion procedure is shown in Figure 4.

### 4.5. Measurement of CK and GOT Activity in CPF

The CPF was collected during reperfusion in the control group, I/R group and S5 + I/R group. The activities of CK and GOT were assayed by the enzymologic method with an Automated Biochemistry Instrument (Hitachi 7060, Hitachi Company, Tokyo, Japan).

### 4.6. Myocardial ERK and P-ERK Detection by Western Blotting

Myocardium stored at −70 °C was weighed, lysed in cell lysis solution measured in the ratio of 1:10 (mass:volume) and then homogenized with ultrasound at 4 °C, and the homogenized solution was centrifuged at 13,000 × *g* for 10 min. The supernatant was then put through electrophoresis, and the gel was loaded with an equivalent amount of mixed protein samples in each pore and then transferred to a nitrocellulose membrane. The membrane was blocked with non-fat milk for 1 h, and primary antibodies were added (ERK1/2 and p-ERK1/2 polyclonal antibodies, diluted to 1:2000) and incubated at 4 °C overnight. Secondary antibodies (goat anti-rabbit monoclonal antibody, diluted to 1:8000) were added to the membrane. The membrane was incubated at room temperature for 1 h, washed by Tween/Tris-buffered salt solution (TTBS) 4 times (10 min each time) and incubated with chemiluminescence for 1 min. After film exposure, development and photographic fixing, protein stripes were scanned with a gel imaging system from AlphaImager (San Leandro, CA, USA), for testing the optical density of the protein stripes.

### 4.7. Statistics

Measurement data are expressed as the mean ± standard deviation (SD). Data were processed by SPSS13.0 (Chicago, IL, USA). One-Way ANOVA followed by least-significance difference (LSD) was used to compare the differences of detected indices among the groups. A paired *t*-test was used to compare the difference between two groups. *p <* 0.05 was considered statistically significant.

## 5. Conclusions

Our study indicated that SO_2_ preconditioning could protect myocardial function and injury by suppressing the over-activated ERK phosphorylation during I/R in isolated perfused rat hearts.

## Figures and Tables

**Table 1:** The activities of creatine kinase (CK) and glutamic-oxaloacetic transaminase (GOT) in the coronary perfusion fluid.

Group	*n*	CK (U/L)	GOT (U/L)
Control	8	3.50 ± 1.85	3.25 ± 0.71
I/R	8	14.33 ± 8.34 ##	6.50 ± 1.88 ##
S5 + I/R	8	6.71 ± 4.23 *	3.71 ± 1.25 **

## *p* < 0.01, *vs.* control group;

** *p* < 0.01;

* *p* < 0.05 *vs.* I/R group.
